# The sublingual use of atropine in the treatment of clozapine‐induced sialorrhea: A systematic review

**DOI:** 10.1002/ccr3.2431

**Published:** 2019-09-27

**Authors:** Thomas Van der Poorten, Marc De Hert

**Affiliations:** ^1^ UPC KU Leuven Kortenberg Belgium; ^2^ Department Of Neurosciences KU Leuven Kortenberg Belgium; ^3^ University of Antwerp Antwerp Belgium

**Keywords:** atropine, clozapine, sialorrhea, sublingual

## Abstract

Clozapine is considered the golden standard in the treatment of therapy‐resistant schizophrenia; however, it associated with bothersome side effects such as sialorrhea. Current evidence suggests that the sublingual use of atropine seems to be safe and effective and could be considered as a first‐line treatment of clozapine‐induced sialorrhea.

## Graphical abstract

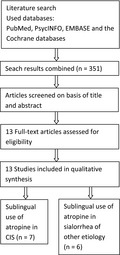

**Limitations**

With respect to the treatment of CIS, only case reports and case series were found, implying a major risk of publication bias. Despite the overwhelmingly positive results, a need for randomized clinical trials remains.

**Summations**

Despite its anecdotal clinical use, a moderate amount of case reports suggest that sublingual atropine is an effective, safe treatment with few side effects in the treatment of CIS.

## 1 INTRODUCTION

There is no doubt that clozapine has taken an important place in the management of treatment‐resistant schizophrenia and can even be seen as the golden standard in treatment‐resistant schizophrenia.^1^, ^2^, ^3^ However, clozapine is associated with a number severe side effects including agranulocytosis, myocarditis, seizures, or sedation. Blood tests are regularly indicated for the determination of leukocytosis and clozapine level. In spite of this, overall mortality in treatment with this drug is lower than with other first‐ or second‐generation antipsychotics.^4^ An irrational fear amongst prescribing psychiatrists seems to play a clinically relevant role in withholding or delaying the initiation of clozapine treatment, unnecessarily prolonging the patients’ suffering.^5^

A total of 30% to even 92%^6^, ^7^of patients taking clozapine are likely to experience an increase in salivation or even excessive salivation or sialorrhea. Despite its occurrence during the day, clozapine‐induced sialorrhea (CIS) is usually the most marked and noticeable at night, usually noticeable by the so‐called “wet pillow sign”.^8^

Both medical and psychosocial adverse effects are associated with sialorrhea. Irritation and maceration can occur at the level of the chin and perioral skin, as well as cheilitis. In combination with the sedative effect of clozapine, aspiration of the oropharyngeal contents, aspiration pneumonia,^9^and even suffocation may occur.^10^ Chronic sleeping problems can occur because of repetitive throat clearing, clozapine‐induced parotitis has also been reported in the past.^11^ At a psychosocial level, wet clothing and the associated odor, as well as speech difficulties and drooling, can have a stigmatizing effect, leading to reduced self‐esteem, social isolation, and ultimately leading to a reduced quality of life.^8^ For instance, more than half of the patients report that CIS has an impact on their quality of life.^7^ These adverse effects may ultimately lead to noncompliance and even discontinuation of clozapine.^12^ This is further complicated by the fact that there is no long‐acting formula of clozapine available to date.

Validated scales such as the Nocturnal Hypersalivation Rating Scale (NHRS) and the Drooling Severity and Frequency Scale (DSF)^7^ can be used. Objective methods such as the measurement of the diameter of the saliva on a patch on the pillow^6^ or weighing dental cotton rolls^13^ can also be used to quantify the severity of CIS. The Drooling Impact Scale (DIS) is often used in children with developmental problems.^14^

Two mechanisms for CIS are suggested: On one hand, the stimulation of the M4 muscarinic receptors and blockade of the α2 receptors by clozapine at the level of the submandibular salivary glands could explain the increased salivation; on the other hand, reduced laryngeal peristalsis^8^ could contribute to the phenomenon. An association was found between the adrenoreceptor alpha 2A gene (ADRA2A) and CIS (*P* = .02^15^), the α2 receptors also appear to be involved in the swallowing movement‐respiratory coordination.^16^ A recent article suggests CIS could be linked to M1‐receptor agonism by clozapine's metabolite norclozapine, a model derived from the effect pirenzepine being a selective M1‐agonist.^17^

Atropine is a racemic mixture of d‐hyoscyamine and l‐hyoscyamine, where the latter is responsible for the most of the physiological effects. It is a competitive antagonist of the muscarinic receptors, thereby inhibiting the effect of acetylcholine, a major neurotransmitter of the parasympathetic nervous system, also innervating the submandibular glands^18^ For example, atropine is used to prevent or treat cholinergic effects during operations by excessive vagal stimulation, or to slow down the unwanted muscarinic effects of drugs.^19^ Orally administered atropine appears safe and well tolerated up to doses of 0.03mg/ kg. The duration of sublingual atropine is probably close to orally and intramuscularly administered atropine: up to 4 hours.^20^, ^21^, ^22^ The effect of atropine on sialorrhea can theoretically be explained by its action as a competitive antagonist of acetylcholine on the muscarinic receptors in the salivary glands.

Various systemic treatment options are available for CIS (anticholinergic medication, α2‐agontists, scopolamine, pirenzepine, and amisulpride) but are generally of little use because of their limited effectiveness or side effects profile. Reviews of the various treatments claim to date that the sample size is too small or that the studies are of insufficient quality to give clinical practice a clear direction.^6^ Furthermore, several lines of evidence indicate that anticholinergic agents may contribute to cognitive deficits in schizophrenic patients, which are a critical determinant of functional outcome.^23^ Other than with clozapine, sialorrhea is also seen to lesser extent with other antipsychotics such as risperidone.^24^ One systematic review examining the effect of anticholinergic medication for nonclozapine neuroleptic‐induced hypersalivation turned out to be an “empty” review as all screened articles were excluded.^25^ Other medication groups associated with drooling are direct and indirect cholinergic agonists that are used to treat dementia of the Alzheimer type and myasthenia gravis.^26^

Two patients of the author, who were successfully treated of CIS by the sublingual use of atropine, gave rise to this systematic review. The aim of this systematic review is to gather the existing evidence for the effectiveness of sublingual application of atropine in reducing or resolving of CIS, as well as looking into the safety, side effects, and dosage of sublingually administered atropine. We also looked at the sublingual use of atropine in sialorrhea of other etiology to get a clearer picture on the effectivity, safety, side effects, and dosage.

## 2 MATERIALS AND METHODS

**Figure 1:**
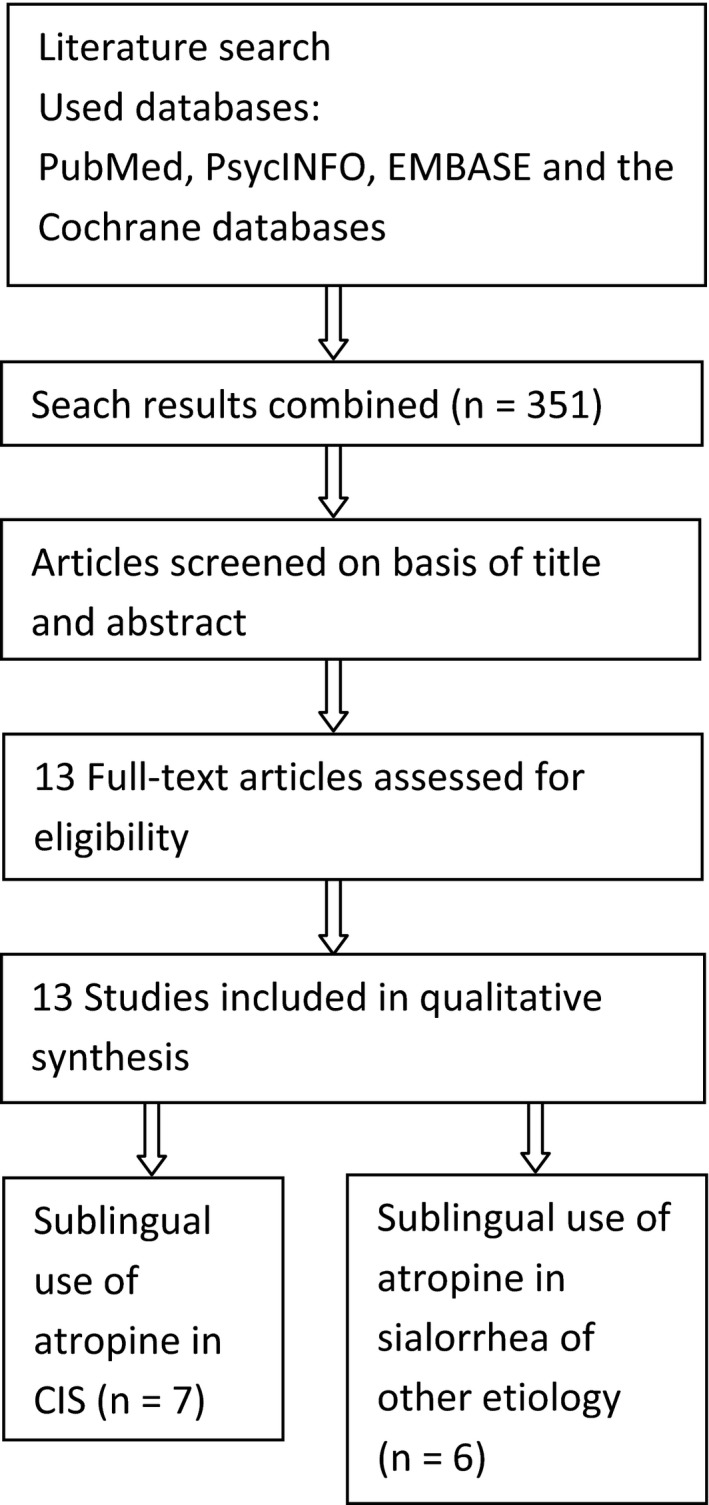
Methods of research using PRISMA

Two independent authors searched MEDLINE/PubMed, PsycINFO, EMBASE, and the Cochrane databases, from their respective inception dates until 1 june 2018 without restriction for language, date of publication, and study design, for the keywords “sialorrhea,” “hypersalivation,” “clozapine” and “atropine.” A first search was performed with all three of the aforementioned keywords, a second search was performed with only “sialorrhea” and “atropine,” to investigate the sublingual use of atropine in sialorrhea of other etiologies (Figure 1). Articles reporting the effect of local use of atropine in the treatment of CIS and sialorrhea of other etiologies were included. An unpublished case series conducted in our center was also included in the results. The reports were published between 1991 and September 2018.

## 3 RESULTS

A total number of 13 articles were found to be eligible for the systematic review. Regarding the local treatment with atropine for CIS, 7 articles were found, no randomized clinical trials, no open label, or retrospective studies were found. The results consisted entirely of case reports and case series (2 and 5 articles respectively, and one unpublished case series in our center). As for the treatment with local atropine in sialorrhea of another etiology, six studies were found, of which only one randomized clinical trial, two pilot studies, one open label study, and two case reports.

### 3.1 CIS treated with local atropine

#### 3.1.1 Case reports

Sharma^27^ describes a patient that achieved a reduction and ultimately a complete resolution of CIS with the use of two drops of atropine 1% twice a day after 2 weeks. She experienced no significant side effects.

Comley et al^28^ reported one patient with schizophrenia that showed a complete resolution of the sialorrhea that was bothersome mainly during the day that after the use of 1 or 2 eye drops of sublingual atropine 1%. They did not mention any side effects.

Finally, Mustapha^29^ published a report on one patient who, despite hyoscine, continued to suffer from severe CID, which had ceased with 1‐3 drops of sublingual atropine 1%, allowing the clozapine levels to be adjusted. However, the CIS did not return after discontinuation of atropine, meaning it could have been a temporary side effect, besides the fact that the treatment was in combination with hyoscine.

#### 3.1.2 Case series

Tessier et al^30^ mention ten patients treated with atropine eye drops that were switched to a nasal spray (ipratropium bromide 0.03%, structurally related to atropine). This form is described as being equally effective at a frequency of 1 to 2 sprays per day. The greater simplicity of use and the longer duration of action were described as an advantage, but a bothersome taste was noted as a possible drawback.

Antonello et al^31^ discuss three patients with severe sialorrhea in his case series, that had an immediate improvement with the administration of one drop of atropine 1% administered sublingually at bedtime. The patients experienced no side effects. They used a “top‐up method” in which one drop was dissolved in glass of water to counter a possible recurrence of salivation during night.

Furthermore, Santana^32^ describes three patients where a complete resolution of the sialorrhea could be observed within 1 week with 1 to 2 drops per night of sublingually administered atropine 1%. This also led to improvements in sleep quality, adherence to clozapine, and no systemic anticholinergic effects were reported. One patient suffered from xerostomia at a dosage of three drops per night.

Leung^33^ describes three patients with a complicated treatment of CIS with sublingual atropine: One disorganized patient emptied the whole bottle, one patient no longer had sialorrhea when stopping atropine after 2 months, and a patient was accidentally given the product in his eye, which impaired his vision during five days.

#### 3.1.3 Own case reports

In our center, we treated two patients with schizophrenia being treated with clozapine that with 1 or 2 sublingually administered atropine eye drops 1% at night showed an almost immediate complete resolution of their nocturnal salivation and showed no side effects.

### 3.2 Sialorrhea of other etiology treated with sublingual atropine

#### 3.2.1 Case reports

A case report^34^ describes sialorrhea in a patient with severe head trauma who was treated with atropine drops, with a 50% reduction of secretion, intraoral, and oropharyngeal accumulation of saliva and had negligible side effects. The exact dose way of measuring the sialorrhea and the exact nature of side effects could not be found.

Another case report from Rapoport et al^35^ describing a child admitted in palliative care with metachromatic leukodystrophy showed that one atropine drop 0.5% sublingually (0.25 mg every 6 hours) had a beneficial effect within 15‐30 minutes, the need of aspiration was greatly reduced and the oxygen saturation improved. The cardiac monitor showed no significant tachycardia, no other anticholinergic side effects were reported.

#### 3.2.2 Open label trial

Dias^36^ investigated 25 children with cerebral palsy, with a statistically significant improvement (measured with the Drooling Impact Scale) before and after treatment with atropine. This was administered sublingually at a concentration of 0.5% 3× a day (with 6 hours interval). There was a low frequency of side effects compared with other anticholinergics.

#### 3.2.3 Pilot studies

A pilot study of Hyson^37^ describing 6 Parkinson's patients and one patient with progressive supranuclear palsy showed a subjective and objective statistically significant reduction in salivation with atropine administered sublingually, measured on a Likert scale (from 1 to 5) and by weighing dental cotton wool rolls. One patient developed a delirium but could also be explained by a concurrent bladder infection; two patients experienced an increase of hallucinations but had concealed the fact that they had those before the start of the study (as this was an exclusion criterion).

Another pilot study about children with sialorrhea (n = 11^38^) with various underlying disorders (including cerebral palsy, epilepsy, and autism) showed a significant reduction in salivation after sublingual administration of atropine eye drops 1‐2× a day, by weighing dental cotton wool rolls and measurement on a visual analogue scale or VAS with the parents). No irreversible adverse reactions were noted, xerostomia occurred in 3 children and some parents reported difficulties with the administration.

### 3.3 Randomized clinical trials

De Simone^39^ performed an RCT in 22 palliative care patients with cancer of the upper gastrointestinal tract, in a prospective randomized placebo‐controlled cross‐over trial. However, the study failed to confirm the effectiveness of atropine sublingually (two drops or 0.5 mg every 6 hours) over placebo in this population. In spite of this, the participants showed improvements on all outcome measures (including sialorrhea). Given the limited sample size, the heterogeneous response and inaccurate measurement (only VAS was used to measure the salivation), the results of this RCT should not be considered as definitive.^33^

## 4 DISCUSSION

A total of 24 patients with CIS were reported, of whom 21 responded favorably to sublingually administered atropine, including two patients who were successfully treated at our center. Leung^33^ is the first to mention possible complications of the treatment that occurred in three of his patients. A total of 67 patients with sialorrhea of other etiology treated with atropine locally were studied sofar with generally a favorable outcome, including one RCT reporting on 22 patients in palliative care.

The fact that the suggested treatment with atropine is administered locally can be seen as an important benefit of this treatment. Although it could have a potentially additive anticholinergic effect due to systemic resorption of clozapine, no reporting of symptoms pointing in this direction has been made yet, even in case of an accidental overdosing.^33^ This is important given the fact that patients may already experience anticholinergic side effects such as constipation, urinary retention, cardiac arrhythmias due to the use of clozapine and may also have cognitive problems due to their disease,^40^ which could on its turn increase due to systemic therapy against the sialorrhea.^23^ Cardiac monitoring in one palliative child treated with a single drop of atropine 0.5% administered sublingually showed no significant tachycardia.^35^ The method is cheap, has a fast onset of action, with in some cases even effect after 15 to 30 minutes.^35^ A nasal spray with ipratropium bromide would have the additional advantage of being more user‐friendly and having a longer duration of action.^30^ Stroup^41^ suggests anticholinergic drops (ipratropium bromide or atropine) as a second‐line treatment, after trying sugarless gum during the day or a towel over the pillow at night.

Possible side effects in the reviewed literature are the occurrence of xerostomia at higher dosages: with one patient with CIS this occurred with three drops^32^ and in 3 out of 11 children studied by Norderyd^38^ taking only 1 to 2 doses per day. The unpleasant taste and at times, the too short duration of action with reoccurrence of salivation in the early morning, which on its turn can be managed by the “top‐up method”.^31^ Some patients experience difficulty in administering the drops in a correct amount.^36^

Some safety concerns include the fact that more disorganized patients or distracted staff members have been reported to accidentally administer the drops in the eyes of the patient, an accidental overdose (as with any product) is also possible.^33^ Some parents of children with sialorrhea treated with atropine sublingually experienced some difficulties in administering it.^38^ Accidental overdoses seem to be more easy with eyedrops than with nose sprays^30^

The posology of atropine eye drops 1% in CIS usually limited 1 to 2 drops 1 to 2× a day. In sialorrhea of other etiology, a more frequent administration with 1 to 2 drops every 6 hours, which seems to make sense in the light of the pharmacokinetics of atropine^20^, ^21^, ^22^

From the case reports and case series (n = 24) reviewed, the authors conclude the sublingual administration of atropine appears to be a simple, effective, safe, and promising treatment for CIS, although Leung^33^ reminds us to be mindful of accidental overdosing and ocular administration. To date, 67 patients with sialorrhea from another etiology were treated with atropine effectively, with the exception of one methodologically weaker RCT, and with few side effects. Despite a lack of randomized controlled studies to clearly inform clinical practice, the reviewed articles suggest that sublingual atropine in the management of CIS is a useful intervention. A recent paper published by Stroup^41^ even suggested it as a second‐line treatment, after trying sugarless gum and a towel over the pillow.

There are several limitations in the literature published up to this date. So far, only case reports and case series have been published on the local treatment with atropine for CIS. The primary outcome measure is not recorded in an accurate way despite the availability of subjective and objective measurement instruments such as the NHRS, the DSFS, the DIS or the diameter of the salivary fluid, a patch on the pillow,^6^, ^7^ or weighing intra‐orally placed dental rolls.^13^ Furthermore, with the exception of one case series, little attention is paid to the systematic questioning of symptoms that may indicate systemic absorption of atropine. Besides, due to the fact that only case reports and case series have been published, the risk of publication bias or placebo effect cannot be overlooked. Moreover, comparative efficacy of sublingual atropine to the existing treatments for CIS cannot be evaluated. Suggestions for further research include performing large‐scaled controlled studies with adequate measuring instruments including dental rolls and systematic evaluation of symptoms indicating systemic absorption of atropine, to have a better view on this promising treatment of CIS.

## CONFLICT OF INTEREST

None declared.

## AUTHOR CONTRIBUTIONS

Thomas VAN DER POORTEN: involved in reviewing of literature and contributed with own case reports. Marc DE HERT: reviewed the literature and reviewed and advised on research.

## References

[1] Tandon R , Nasrallah HA , Keshavan MS . Schizophrenia, “Just the Facts” 5. Treatment and prevention past, present, and future. Schizophr Res. 2010;122(1–3):1‐23. 20655178 10.1016/j.schres.2010.05.025

[2] Samara MT , Dold M , Gianatsi M , et al. Efficacy, acceptability, and tolerability of antipsychotics in treatment‐resistant schizophrenia: a network meta‐analysis. JAMA psychiatry. 2016;73(3):199‐210. 26842482 10.1001/jamapsychiatry.2015.2955

[3] Lewis SW , Barnes TR , Davies L , et al. Randomized controlled trial of effect of prescription of clozapine versus other second‐generation antipsychotic drugs in resistant schizophrenia. Schizophr Bull. 2006;32(4):715‐723. 16540702 10.1093/schbul/sbj067 PMC2632262

[4] Tiihonen J , Lönnqvist J , Wahlbeck K , et al. 11‐year follow‐up of mortality in patients with schizophrenia: a population‐based cohort study (FIN11 study). The Lancet. 2009;374(9690):620‐627. 10.1016/S0140-6736(09)60742-X 19595447

[5] Cohen D . Prescribers fear as a major side‐effect of clozapine. Acta Psychiatr Scand. 2014;130(2):154‐155. 24841141 10.1111/acps.12294

[6] Syed R , Au K , Cahill C , et al. Pharmacological interventions for clozapine‐induced hypersalivation. Cochrane Database Syst Rev. 2008;3:CD005579. 10.1002/14651858.CD005579.pub2 PMC4160791 18646130

[7] Maher S , Cunningham A , O'Callaghan N , et al. Clozapine‐induced hypersalivation: an estimate of prevalence, severity and impact on quality of life. Ther Adv Psychopharmacol. 2016;6(3):178‐184. 27354906 10.1177/2045125316641019 PMC4910403

[8] Praharaj SK , Arora M , Gandotra S . Clozapine‐induced sialorrhea: pathophysiology and management strategies. Psychopharmacology. 2006;185(3):265‐273. 16514524 10.1007/s00213-005-0248-4

[9] Saenger RC , Finch TH , Francois D . Aspiration pneumonia due to clozapine‐induced sialorrhea. Clin Schizophr Relat Psychoses. 2016;9(4):170‐172. 23773887

[10] Hinkes R , Quesada TV , Currier MB , Gonzalez‐Blanco M . Aspiration pneumonia possibly secondary to clozapine‐induced sialorrhea. J Clin Psychopharmacol. 1996;16(6):462‐463. 8959476 10.1097/00004714-199612000-00013

[11] Brodkin ES , Pelton GH , Price LH . Treatment of clozapine‐induced parotid gland swelling. Am J Psychiatry. 1996;153(3):445. 10.1176/ajp.153.3.445a 8610845

[12] Rogers DP , Shramko JK . Therapeutic options in the treatment of clozapine‐induced sialorrhea. Pharmacotherapy. 2000;20(9):1092‐1095. 10999502 10.1592/phco.20.13.1092.35036

[13] Chou KL , Evatt M , Hinson V , Kompoliti K . Sialorrhea in Parkinson's disease: a review. Mov Disord. 2007;22(16):2306‐2313. 17659637 10.1002/mds.21646

[14] Reid SM , Johnson HM , Reddihough DS . The drooling impact scale: a measure of the impact of drooling in children with developmental disabilities. Dev Med Child Neurol. 2010;52(2):e23‐e28. 19843155 10.1111/j.1469-8749.2009.03519.x

[15] Solismaa A , Kampman O , Seppälä N , et al. Polymorphism in alpha 2A adrenergic receptor gene is associated with sialorrhea in schizophrenia patients on clozapine treatment. Human Psychopharmacology: Clinical and Experimental. 2014;29(4):336‐341. 25163438 10.1002/hup.2408

[16] Yamanishi T , Takao K , Koizumi H , et al. α2‐Adrenoceptors coordinate swallowing and respiration. J Dent Res. 2010;89(3):258‐263. 20139342 10.1177/0022034509360312

[17] Meyer J . Clozapine‐Induced Sialorrhea: Why Is It Important and How Should You Manage It? [online] Psychopharmacology Institute. Available at: https://psychopharmacologyinstitute.com/antipsychotics/clozapine-induced-sialorrhea-important-manage/. [Accessed 12 Aug. 2018].

[18] Carlson GW . The salivary glands: embryology, anatomy, and surgical applications. Surgical Clinics. 2000;80(1):261‐273. 10685152 10.1016/s0039-6109(05)70405-9

[19] National Center for Biotechnology Information . PubChem Compound Database; CID=5927, https://pubchem.ncbi.nlm.nih.gov/compound/5927. Accessed July 31, 2018.

[20] Saarnivaara L , Kautto UM , Iisalo E , Pihlajamäki K . Comparison of pharmacokinetic and pharmacodynamic parameters following oral or intramuscular atropine in children. Acta Anaesthesiol Scand. 1985;29(5):529‐536. 4036539 10.1111/j.1399-6576.1985.tb02248.x

[21] Gervais H . Plasma concentration following oral and intramuscular atropine in children and their clinical effects. Journal of Pediatric Anesthesia. 1997;7(1):13–18. 9041569 10.1046/j.1460-9592.1997.d01-40.x

[22] Takemoto CK , Hodding JH , Kraus DM . Lexi‐Comp's Pediatric Dosage Handbook. Hudson: Lexi-Comp; 2004.

[23] Liang CS , Ho PS , Shen LJ , Lee WK , Yang FW , Chiang KT . Comparison of the efficacy and impact on cognition of glycopyrrolate and biperiden for clozapine‐induced sialorrhea in schizophrenic patients: a randomized, double‐blind, crossover study. Schizophr Res. 2010;119(1–3):138‐144. 20299191 10.1016/j.schres.2010.02.1060

[24] Gajwani P , Franco‐Bronson K , Tesar GE . Risperidone‐induced sialorrhea. Psychosomatics. 2001;42(3):276. 10.1176/appi.psy.42.3.276 11351120

[25] Essali A , Rihawi A , Altujjar M , Alhafez B , Tarboush A , Hasan N. A. . (2013). Anticholinergic medication for non‐clozapine neuroleptic‐induced hypersalivation in people with schizophrenia. Cochrane Database of Systematic Reviews, (12). 10.1002/14651858.CD009546.pub2 PMC11357688 24353163

[26] Freudenreich O . Drug‐induced sialorrhea. Drugs of Today. 2005;41(6):411‐418. 16110348 10.1358/dot.2005.41.6.893628

[27] Sharma A , Ramaswamy S , Dahl E , Dewan V . Intraoral application of atropine sulfate ophthalmic solution for clozapine‐induced sialorrhea. Ann Pharmacother. 2004;38(9):1538‐1538. 10.1345/aph.1E077 15252196

[28] Comley C , Galletly C , Ash D . Use of atropine eye drops for clozapine induced hypersalivation. Aust N Z J Psychiatry. 2000;34(6):1033‐1034. 11127617 10.1177/000486740003400102

[29] Mustafa FA . Sublingual atropine for the treatment of severe and hyoscine‐resistant clozapine‐induced sialorrhea. African J Psychiatry. 2013;16(4):236‐242. 10.4314/ajpsy.v16i4.32 24051562

[30] Tessier P , Antonello C . Clozapine and sialorrhea: update. J Psychiatry Neurosci. 2001;26(3):253. PMC1408292 11394196

[31] Antonello C , Tessier P . Clozapine and sialorrhea: a new intervention for this bothersome and potentially dangerous side effect. J Psychiatry Neurosci. 1999;24(3):250. 10354662 PMC1189018

[32] Santana T , Capurso NA , Ranganathan M , Yoon G . Sublingual atropine in the treatment of clozapine‐induced sialorrhea. Schizophr Res. 2017;182:144‐145. 27816262 10.1016/j.schres.2016.10.039

[33] Leung JG , Schak KM . Potential problems surrounding the use of sublingually administered ophthalmic atropine for sialorrhea. Schizophr Res. 2017;185:202‐203. 28043733 10.1016/j.schres.2016.12.028

[34] Dworkin JP , Nadal JC . Nonsurgical treatment of drooling in a patient with closed head injury and severe dysarthria. Dysphagia. 1991;6(1):40‐49. 1884637 10.1007/BF02503462

[35] Rapoport A . Sublingual atropine drops for the treatment of pediatric sialorrhea. J Pain Symptom Manage. 2010;40(5):783‐788. 20541902 10.1016/j.jpainsymman.2010.02.007

[36] Dias B , Fernandes AR , Maia Filho H . Treatment of drooling with sublingual atropine sulfate in children and adolescents with cerebral palsy. Arq Neuropsiquiatr. 2017;75(5):282‐287. 28591387 10.1590/0004-282X20170033

[37] Hyson HC , Johnson AM , Jog MS . Sublingual atropine for sialorrhea secondary to parkinsonism: a pilot study. Mov Disord. 2002;17(6):1318‐1320. 12465075 10.1002/mds.10276

[38] Norderyd J , Graf J , Marcusson A , et al. Sublingual administration of atropine eyedrops in children with excessive drooling ‐ a pilot study. Int J Paediatr Dent. 2017;27(1):22‐29. 26708211 10.1111/ipd.12219 PMC5324542

[39] De Simone GG , Eisenchlas JH , Junin M , Pereyra F , Brizuela R . Atropine drops for drooling: a randomized controlled trial. Palliat Med. 2006;20(7):665‐671. 17060265 10.1177/0269216306071702

[40] Green MF . Cognitive impairment and functional outcome in schizophrenia and bipolar disorder. J Clin Psychiatry. 2006;67:3‐8. 16965182

[41] Stroup TS , Gray N . Management of common adverse effects of antipsychotic medications. World Psychiatry. 2018;17(3):341‐356. 30192094 10.1002/wps.20567 PMC6127750

